# Finished Genome Sequence of a Polyurethane-Degrading Pseudomonas Isolate

**DOI:** 10.1128/genomeA.00084-18

**Published:** 2018-03-01

**Authors:** Blake W. Stamps, Sandra Zingarelli, Chia-Suei Hung, Carrie A. Drake, Vanessa A. Varaljay, Bradley S. Stevenson, Wendy J. Crookes-Goodson

**Affiliations:** a Department of Microbiology and Plant Biology, University of Oklahoma, Norman, Oklahoma, USA; b UES, Inc., Dayton, Ohio, USA; c Soft Matter Materials Branch, Materials and Manufacturing Directorate, Air Force Research Laboratory, Wright-Patterson AFB, Ohio, USA

## Abstract

Pseudomonas sp. strain WP001 is a laboratory isolate capable of polyurethane polymer degradation and harbors a predicted lipase precursor gene. The genome of strain WP001 is 6.15 Mb in size and is composed of seven scaffolds with a G+C content of 60.54%. Strain WP001 is closely related to Pseudomonas fluorescens based on ribosomal DNA comparisons.

## GENOME ANNOUNCEMENT

Bacteria within the genus Pseudomonas are metabolically diverse and are of both environmental and industrial importance. Members of the genus are known to degrade hydrocarbons (1) and other complex carbon substrates (2). Specifically, Pseudomonas isolates, including near relatives of the sequenced isolate, are capable of polyurethane degradation (3, 4). Polyurethanes comprise a large amount of polymer waste, and thus isolates capable of polyurethane degradation represent an important area for bioremediation research. A Pseudomonas isolate, strain WP001, was obtained from a laboratory coculture. The genome of WP001 was sequenced and is reported here, in part because it was able to degrade the polyurethane Impranil DLN.

Cellular biomass of Pseudomonas sp. strain WP001 was extracted from a culture using the Qiagen blood and cell culture DNA minikit (Qiagen, Inc., Germantown, MD, USA) to produce sufficient quantities of DNA for genomic sequencing on a single Pacific Biosciences RS II single-molecule real-time (SMRT) cell using P6-C4 sequencing chemistry. Assembly was carried out with Canu (5) version 1.6 using default parameters, resulting in an estimated genome size of 6 Mb. Genome assembly statistics were computed using Quast (6). The assembly was evaluated for completeness and contamination using CheckM (7) to ensure that the assembly was free of any contaminating sequences. An initial annotation was produced using Prokka (8), while the final reported annotation was produced using the NCBI Prokaryotic Genome Annotation Pipeline (9).

Strain WP001 contained seven scaffolds after assembly. The *N*_50_ and total genome lengths were 4.1 Mb and 6.15 Mb, respectively, and the G+C content was 60.54%. The genome was estimated to be 99.93% complete, with 1,032 marker genes represented once and 11 single-copy marker genes represented twice, placing its expected lineage within the genus Pseudomonas. Six complete (1,532-bp) 16S rRNAs were recovered within the genome and identified as being most closely related (99% nucleotide similarity) to a cluster of species, including P. fluorescens, which is known to be capable of polyurethane degradation (10). A predicted lipase precursor gene with 91% amino acid similarity to a known polyurethanase (PueA) was also identified in the genome.

The genome described here is of particular informational value and will help further research on polymer degradation and metabolism. Future work will leverage the data of this and other genomes to develop a better understanding of the underlying molecular mechanisms of polyurethane degradation.

### Accession number(s).

This whole-genome shotgun sequencing project has been deposited in GenBank under the accession number PGRY00000000.
